# Real-time vascular mechanosensation through *ex vivo* artery perfusion

**DOI:** 10.1186/1480-9222-16-6

**Published:** 2014-03-31

**Authors:** Rahul M Prasad, Xingjian Jin, Wissam A AbouAlaiwi, Surya M Nauli

**Affiliations:** 1 College of Pharmacy and Pharmaceutical Sciences, The University of Toledo, Toledo, OH, USA; 2 College of Medicine and Life Science, The University of Toledo, Toledo, OH, USA; 3 Department of Pharmacology; MS 1015, Health Education Building; Room 274, The University of Toledo, 3000 Arlington Ave, Toledo, OH 43614, USA

## Abstract

**Background:**

Cell-based perfusion studies have provided great insight into fluid-sensing mechanisms, such as primary cilia in the renal and vascular systems. However, the intrinsic limitations of *in vitro* cell culture, such as the inability to reflect cellular organization within tissues, has distanced observed paradigms from possible clinical developments. Here we describe a protocol that applies *ex vivo* artery perfusion and calcium imaging to observe real-time cellular responses to fluid-shear stress.

**Results:**

Through our *ex vivo* artery perfusion method, we were able to simulate physiological flow and initiate distinct fluid shear stress mechanosensory responses, as well as induced acetylcholine responses in mouse aortic tissue. The observed calcium profiles confirm results found through previous *in vitro* cell culture experiments. The overall procedure, including dissection, sample preparation and perfusion, takes around 3 hours to complete.

**Conclusion:**

Through our unique method, we are able to induce laminar flow within intact mouse aortic tissue and illicit subsequent cellular responses. This method of *ex vivo* artery perfusion provides the opportunity to bridge the novel findings of *in vitro* studies with subsequent physiological models of fluid-shear stress mechanosensation in vascular tissues.

## Background

Mechanosensation of blood flow is a crucial function of the vascular system. Defects in the ability to adequately sense and respond to blood flow have been linked to several cardiovascular pathologies including hypertension, atherosclerosis, and aneurysms [1]. Through the use of *in vitro* assays, which utilize fluid perfusion of cultured cells, numerous insights have been made into the mechanisms of flow sensation such as the role of the primary cilium, endothelial glycocalyx, cytoskeleton and many others.

Despite breakthroughs in identifying mechanisms of blood flow, the *in vitro* perfusion system remains limited in its application to understanding and treating disease states by their inability to replicate crucial *in vivo* influences. The diversity of cellular populations within blood vessels is an excellent example of this; current cell culture technologies are not capable of emulating the organization of several cell types. The organization of cell-types have been shown to be essential to such functions as remodeling in atherosclerosis models as well as cell-cell communication, as is the case between endothelial cells and vascular smooth muscle cells through nitric oxide [2,3]. In addition to this, the physical structure of vascular tissue remains elusive to *in vitro* approaches. Local hemodynamic forces in vascular tissue, produced by curvatures, lumen diameter, and branch points, have been shown to have profound effects in the form of gene expression and pathogenesis [4]. While *in vitro* studies have been able to simulate and confirm the effects of some of these forces, such as shear stress, *ex vivo* studies remain the most physiologically relevant due to their ability to observe the combined effects of nearly all the fluid forces acting on the cells.

Due to the breath of literature citing intracellular Ca^2+^ as a secondary messenger, the use of fluorescent calcium indicators is chosen as the means of observing endothelial sensory function. Within endothelial cells, intracellular Ca^2+^ has been shown to be vitally involved in the maintenance of tone, nitric oxide synthesis, ion channel regulation, and triggering of growth mechanisms. Intracellular Ca^2+^ has also been established as an read-out of monitoring fluid flow sensation in numerous other cell types including stem cells [5], renal epithelial [6], hepatic epithelial [7], pancreatic duct [8], nodal epithelial [9], dentin [10] , osteoblasts and osteocytes [11,12].

**Figure 1:**
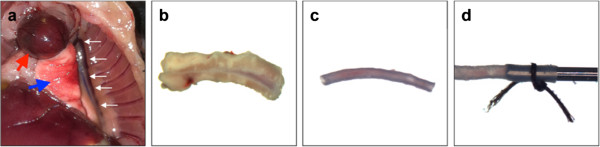
**Dissection, preparation and cannulation of mouse vascular tissue.** In this figure, mouse thoracic aorta is shown as an example. **a**. Mouse thoracic cavity is exposed, and heart (red arrow) is displaced to better reveal the lung (blue arrow) and aorta (white arrows). **b**. After the aorta is dissected out from the mouse, blood is perfused out from the lumen with a 30 gauge needle. The encapsulation of vessel by fat and connective tissue is visible. **c**. Aorta is cleaned by removing the surrounding tissues. **d**. The aorta is then cannulated and tied with silk suture to form a surgeon’s knot.

**Figure 2:**
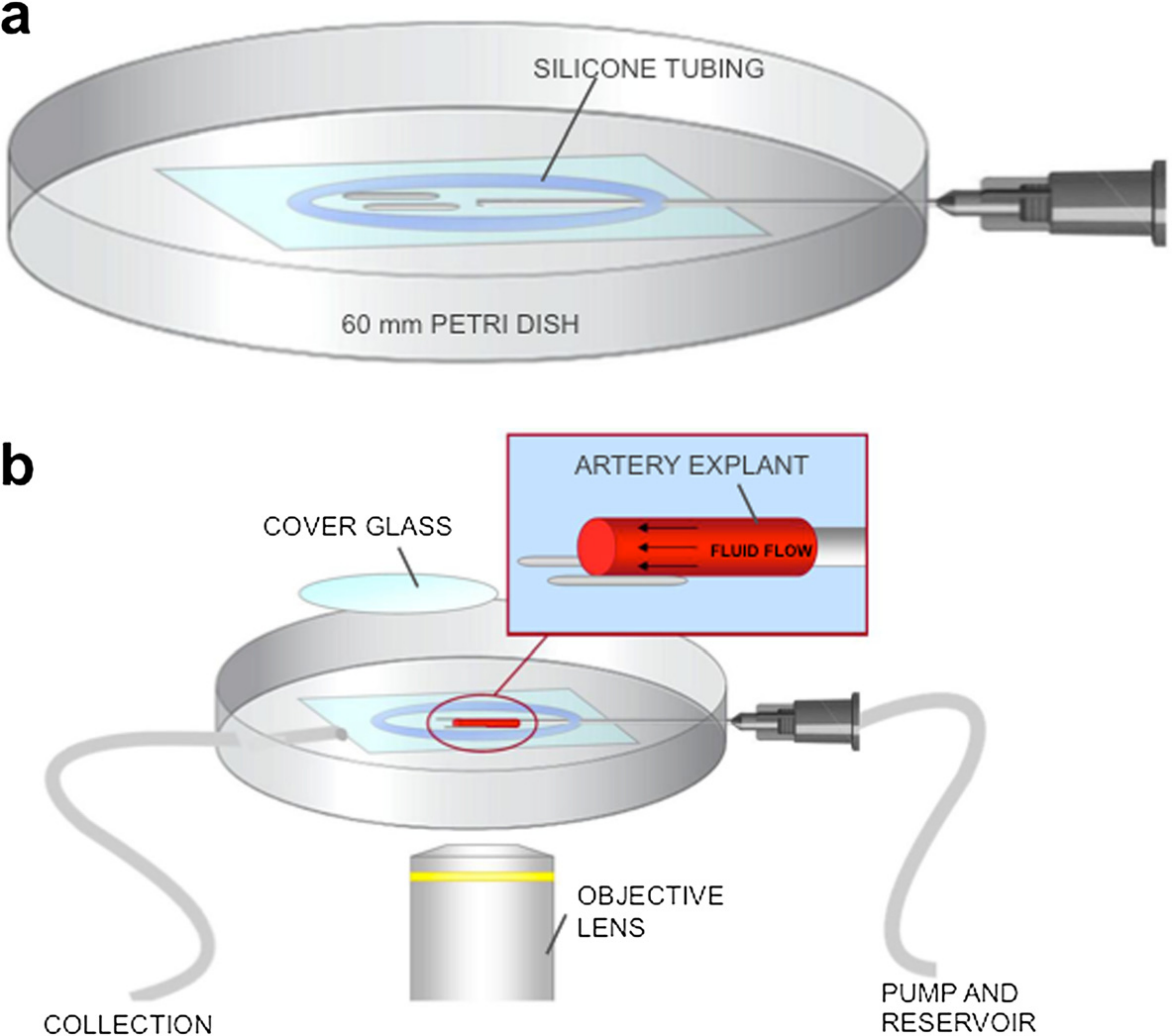
**Schematic representation of perfusion apparatus. a**. An inexpensive apparatus can be made using a 100-mm cell culture dish, a 22-g hypodermic needle, glass capillary tubes, a glass cover slip, silicone tubing, and non-cytotoxic epoxy and silicone glues. **b**. After cannulation to the apparatus, the vessel is covered with a cover glass. The chamber is placed on the microscope and connected with the rest of perfusion system.

**Figure 3:**
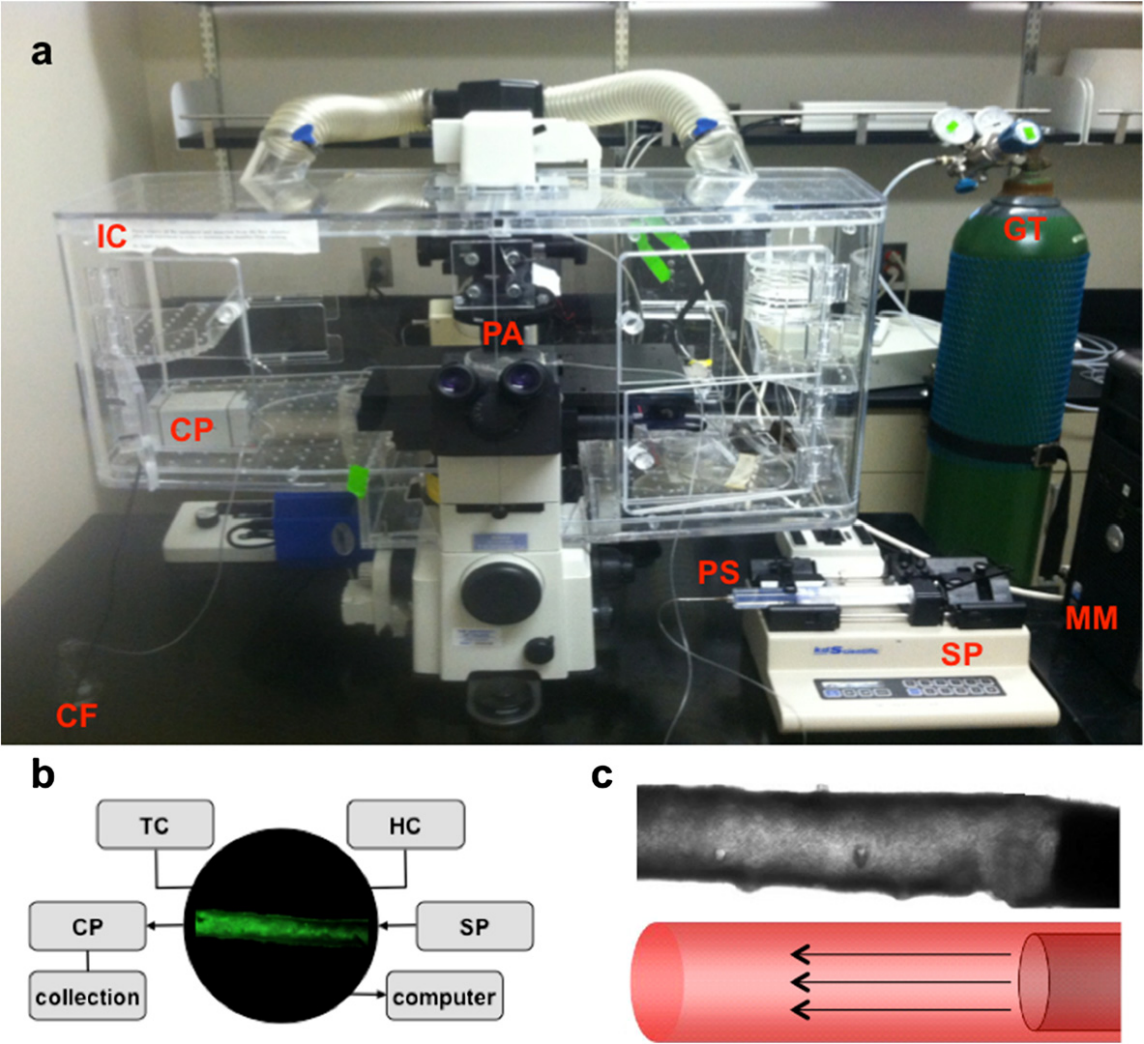
**Overview of experimental system.** An ex vivo perfusion fluid-flow setup is shown to maintain a healthy functional tissue for the duration of experiment. **a**. The setup includes a computer with MetaMorph software (MM), syringe pump (SP), perfusion syringe (PS), perfusion apparatus (PA), collection pump (CP), collection flask (CF), incubation chamber (IC), gas tank (GT). **b**. Not visible in picture is a temperature controller (TC), humidity controller (HC), and computer monitor. Artery segment has a baseline fluorescent after loaded with calcium indicator. **c**. Brightfield image of the artery segment is visualized. As depicted by the diagram below, a perfusion needle is cannulated to the artery to deliver fluid-flow.

In our study, we developed a method for observing fluid-shear stress mechanosensation in mouse aortic explants (Figure 1). Although *ex vivo* perfusion studies have been conducted on vascular tissues, our method provides the novel ability to deliver and induce mechanosensory vascular responses unique to fluid shear stress [13-18]. The experimental method we describe provides the ability to induce laminar flow through a cannulated artery without damaging the endothelia, maintain the artery in a physiologically relevant environment, and clearly visualize the tissue (Figures 2 and 3).

## Results and discussion

**Figure 4:**
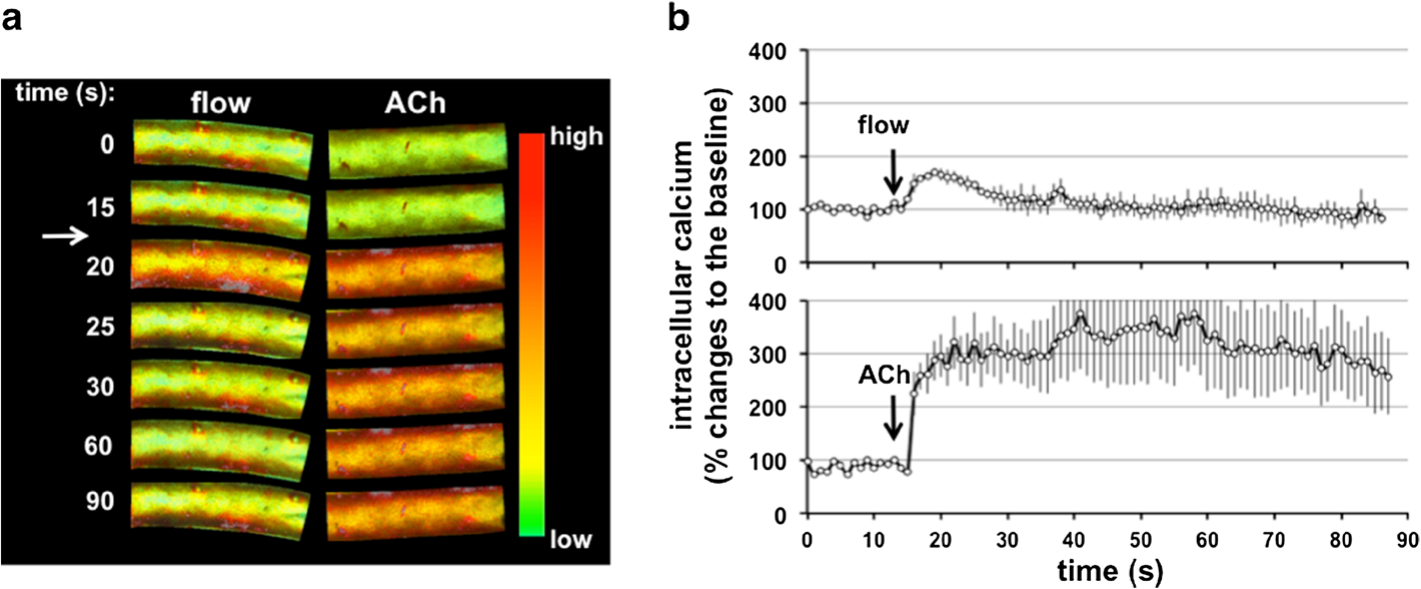
**Distinct calcium responses to fluid-shear stress and a pharmacological agonist.** After loaded with calcium indicator, a segment of artery is imaged under an inverted microscope. Baseline calcium level is recorded for at least 15 seconds (s). **a**. Pseudocolor indicates intracellular calcium level in response to fluid-shear stress in the presence or absence of acetylcholine (ACh). Green to red color indicates low to high calcium level, respectively. **b**. Changes in calcium level in response to flow or acetylcholine are quantified and normalized relative to baseline calcium level. Arrows indicate the initiation of perfusion.

The described methodology allows for the observation of cellular responses to fluid flow. The calcium response profile for induced fluid flow in wild type mouse tissue (Figure 4) is very similar to the extensively characterized profile of endothelial *in vitro* responses [19,20]. This response is characterized by a rapid increase in intracellular calcium, peaking after approximately 5 seconds of induction, and then plateau below the baseline. The similarity of the profiles suggests that the readout is indicative of the shear stress response transduced by endothelial cilia. In addition to the fluid flow response, the profile for tissue induced by acetylcholine again matches those of endothelial *in vitro* responses [19,20]. This response is distinguished in both systems as a rapid increase within 5 seconds, at a slightly higher level than that of fluid flow alone, and a sustained elevation of intracellular calcium. As the response to acetylcholine has been widely characterized, it is used as a control for viability.

Based on lumen diameter, we used a flow rate to induce a shear stress of 7.2 × 10^4^ kg/m/sec^2^, as this has been shown in *in vitro* studies to be the optimal level of endothelial shear stress response [19,20]. We found that at least 3 flow experiments were able to be conducted before the tissue was rendered unviable. Importantly, our set-up confirms that our tissue was in focus throughout our experimental procedure (Additional files 1 and 2: Movies 1 and 2). In addition, the differential interference contrast movies also indicate a stably positioned tissue within our set-up, suggesting no abrupt change in pressure or disruption was observed during our experiment.

While the primary advantage of our method is the ability to observe vascular mechanosensory responses to shear stress, the system can be adapted for other appropriate applications. For example, pressure sensing is a common approach for recording responses through tension changes as a result of vasoconstriction or dilation in blood vessels [21]. In addition, the potential for *ex vivo* perfusion to study the etiology of atherosclerosis has recently arisen [18]. Similar to the use of fluorescence imaging to observe responses of the vascular endothelial or smooth muscle cells, other groups have used fluorescence imaging to observe leukocyte adhesion to the endothelium of perfused vessels – a crucial process in the pathogenesis of atherosclerosis [15,17,18]. Finally, *ex vivo* artery perfusion has also been utilized as a means of drug delivery development [16].

## Conclusion

In order to apply the insights of *in vitro* studies to physiological models, we have developed *ex vivo* artery perfusion technology to allow for the measurement of the cellular response as a result of fluid-shear stress mechanosensation. This approach may serve as the next step in applying *in vitro* findings in a more physiologically relevant manner and in investigating the mechanisms of vascular mechanosensation – a crucial measure towards developing therapeutics for vascular disease.

## Materials

### Reagents

Adult mouse; distilled water; DMEM (Thermo Scientific, cat. no. SH30243.01); Fluo-2 AM (TEFLabs, cat. no. 0222); acetylcholine chloride (Sigma, a2661-25 g); dimethyl sulfoxide (Sigma, d8779); ethanol, 95%, vol/vol (PharmcoAAPER, 111000190); silicone (GE, stock no. GE50090442); epoxy (Henkel, co. no. 1393760).

### Equipment

Sterile dissection instruments: forceps (Roboz Surgical), scissors (Roboz Surgical); culture dishes, 100 mm (Thermo Scientific cat. no. 130182); syringe, 1 ml (BD Luer-Lok, cat. no. 309628); Syringe, 30 mL - Luer (Heinke-Sass-Wolf, cat. no. 4830001000); hypodermic needles, 22 gauge (BD PrecisionGlide, cat. no. 305156); hypodermic needles, 30 gauge (BD PrecisionGlide, cat. no. 305106); sutures 5–0 (Teleflex Medical); microscope slides (Fisherbrand, cat. no. 451000); coverslips, 22 mm × 32 mm (VWR International, cat. no. 831–0134); capillary, outside diameter of 1.5 mm, thickness of 0.2 mm (Kimble, Inc.); humidified, water-jacketed incubator (Binder) at 37°C and 5% CO_2_; dissecting microscope (Zeiss, Stemi SV6); Nikon microscope (Eclipse, TE2000-U); Photometric monochrome camera (Coolsnap EZ 20 MHz); light box (Zeiss); perfusion pump; Nalgene 180 PVC tubing, 1/32 in. I.D., 3/32 in. O.D., 1/32 in. wall (Thermo Scientific, part no. 8000–002); CO_2_ gas tank with regulator.

## Methods

### Reagent setup

Ethanol (70%, vol/vol) was prepared by diluting absolute ethanol in distilled water and store indefinitely at room temperature. 50 μg of Fluo-2 AM was dissolved in 50 μL of dimethyl sulfoxide, distributed into 5 μL aliquots and stored indefinitely at −20°C. A variety of buffers may be used to may be used to bathe and perfuse the tissue samples – we chose HEPES-based DMEM (see Reagents). Buffers were stored at 4°C until use. Before perfusion, the buffer was warmed at 37°C for 30 min. Throughout our experiment, we used DMEM because it has similar mechanic properties as blood plasma. In addition, DMEM acts as Newtonian fluid making it easier in our fluid-shear stress calculation. For pharmacological reagents, a stock solution was made in distilled water and at an appropriate volume to a volume of buffer for the desired concentration. The final solution was loaded into the perfusion syringe prior to treatment. For acetylcholine, we made a 100 mM stock solution, then added 40 μL of the stock to 40 mL of DMEM for a final concentration of 10 μM.

### Equipment setup

Mice were housed in pathogen-free, regulated-humidity conditions and kept in a 12-h light cycle with sterilized chow and water provided. The ‘Three Rs’ (replacement, reduction, refinement) were observed in the design and execution of all experiments. The use of animal cells or tissues was approved by the Animal Care and Use Committee of The University of Toledo. We have complied with all the governmental and institutional guidelines for the care and use of animals within our research program. All dissection instruments were kept in 70% (vol/vol) ethanol during the procedure to maintain sterility.

A Nikon TE2000-U was connected with a photometric monochrome camera (Coolsnap EZ 20 MHz). A high speed excitation wavelength changer for DG4/DG5 system was used to capture at DIC and a fluorescence excitation wavelength of 380 nm, under the control of MetaFluor / MetaMorph software.

### Building the perfusion apparatus

A glass bottom dish was used to make the perfusion apparatus, as the plastic polymers used in other culture dishes interferes with the recording of fluorescence (Figure 2). To make this, a circular hole was made in the center of a 100 mm cell culture dish and Epoxy was used to glue a coverslip to cover the hole (see Materials). Using small diameter silicone tubing (Thermo Scientific Nalgene 180 PVC tubing) a 35 mm closed ring was then formed. The ring was around the glass bottom center of the dish, using silicone. The silicone was left to dry for 2 hours, forming a sealed barrier to prevent leakage. A slit in the culture dish was made, which was wide enough to rest the bevel of the needle on. The tip of a 22 g needle was removed and blunted. The needles was then passed through the slit toward the middle of the ring, piercing through the silicone in the process. The needle was angled at no more than 3 degrees from the bottom of the dish. Using Epoxy, the needle was glued in place, allowing the epoxy to dry for 2 hours. In order to prevent the tissue from swaying during perfusion, two pieces of capillary tube were taped across from the tip of the perfusion needle.

### Collection and preparation of aortic tissue

First, a mouse was euthanized through CO_2_ asphyxiation. As always, all animal experiments complied with national laws and institutional regulations. In a tray with paper towels, the animal was positioned on its back to dissect the aorta. A transverse incision was made to open the abdominal cavity, followed by cutting of the diaphragm. Incisions where then made up the chest wall to the first or the second rib on both sides. A final incision was made along the sternum to expose the heart. The aorta was exposed by displacing the lungs and intestines, and is adhered to the vertebral column through fatty connective tissue. After locating the aorta, the arch of the aorta is decannulated from the heart, brachiocephalic, carotid and subclavian arteries. In a cranial to caudal direction, the thoracic and abdominal aorta was slowly separated from the vertebral column. Finally, the abdominal aorta was decannulated from the distal aorta.

In order to remove intraluminal blood, the tissue was placed in ice cold oxygenated DMEM and slowly perfused with 0.5 mL of DMEM. We found that perfusion must be conducted gently and no more than twice - multiple or high pressure perfusion will damage the endothelium of the vessel and render the tissue unresponsive. After removing the intraluminal blood, fatty connective tissue surrounding the vessel was gently cut away, taking care to prevent large nicks and holes in the tissue.

A 5 microliter aliquot of Fluo-2 calcium dye was diluted with 250 microliters of DMEM in a centrifuge tube. The tissue sample was then placed in centrifuge tube. The centrifuge tube was then incubated for 30 minutes in a CO_2_ incubator. Following incubation, the tissue was carefully removed placed in a centrifuge tube containing 500 microliters of DMEM, and then incubated for 10 minutes.

### Preparing and connecting the perfusion pump

The organ bath chamber of the apparatus was filled with 4 mL of oxygenated warm DMEM, and the aorta sample was placed within in the organ bath. Prior to cannulation of the sample with the perfusion needle, 1 mL of perfusion solution was gently pipeted onto and through the connection port of the perfusion apparatus, thereby preventing air bubbles.

Using microforceps, the aorta was cannulated by sliding the sample over the needle. A small nick was made in the tissue, past the tip of the perfusion needle, in order to relieve the negative pressure inside the tissue that is generated automatically following cannulation. Excessive stretching will trigger stretch activated calcium channels and will ruin the integrity of the sample. After cannulation, a glass coverslip was taped over the organ bath chamber. We found that if a coverslip is not taped, the increase in fluid level as a result of perfusion causes a drastic drop in recorded fluorescence. The apparatus was then placed on the microscope stage. The tissue was left to equilibrate undisturbed in the apparatus for 15 minutes and but no longer than 30.

In order to prepare the perfusion pump, the perfusion tubing was first placed into the perfusion pump. Warmed, oxygenated DMEM was pre-perfused through the clean silicone tubing. The perfusion pump tubing was then connected to the perfusion apparatus and the speed of perfusion was set on the pump.

### Perfusion and real-time fluorescence imaging

Metamorph imaging software was used to record fluorescence levels of the sample. Channels of DIC and GFP (with excitation and emission filters of 400 ~ 430 nm and 508 ~ 520 nm, respectively) were simultaneously captured with 1.07 s per frame. After recording a baseline of at least 15 seconds, the perfusion pump, with speed pre-set, was turned on. After 91 frames, perfusion was stopped. To prevent excess build up of media spilling out of the silicon ring within the perfusion apparatus, an additional pump and tubing can be attached to the apparatus in parallel and outside the ring.

### Data analysis

The captured images were compiled into a sequential stacked file. The background of the perfusion experiment was logged by selecting an identical region of the image plane, where no part of the sample overlapped. The average intensity of the region was logged to Microsoft Excel. Similarly, to analyze the sample, a region of the image plane was selected with only the sample contained. In cases where significant movement of the sample was observed, the region of data collection was appropriately adjusted on a frame by frame basis. The average intensity of the sample region was logged to the same Microsoft Excel file as the background values. The recorded fluorescence values of the background region were subtracted from the values of the tissue sample region. This allowed for fluorescence changes due to noise to be accounted for.

An average was calculated from the first fifteen data points of the new, adjusted values. This value was used as the recorded baseline, as this was the average intensity of the tissue prior to perfusion. The entire data set was then normalized by dividing all of the adjusted data set values by the calculated averaged baseline. The normalized data points were plotted on a graph to produce a representative trace. At least three experiments under each condition – perfusion and treatment with ACh. The data sets were respectively averaged and plotted with error bars indicated standard error.

## Competing interests

The author’s claim no competing financial interests.

## Authors’ contributions

Developing original approach (WAA, SMN), improving and confirming the technique (RMP, XJ), performing experiment (RMP), analyzing data (RMP, XJ), writing the manuscript (RMP, SMN). All authors read and approved the final manuscript.

## Supplementary Material

Additional file 1 **Movie 1.** Flow induced a transient intracellular calcium response in mouse artery *ex vivo*. Differential interference contrast (left) and fluorescence (right) movies were captured simultaneously at 1 frame per second. Number indicates time in second. Color bar indicates pseudocolor range where purple and white represent lowest and highest intracellular calcium levels, respectively. Click here for file

Additional file 2 **Movie 2.** Acetylcholine induced a sustained increase in intracellular calcium in mouse artery *ex vivo*. Differential interference contrast (left) and fluorescence (right) movies were captured simultaneously at 1 frame per second. Number indicates time in second. Color bar indicates pseudocolor range where purple and white represent lowest and highest intracellular calcium levels, respectively. Click here for file
